# Identifying Chinese Medicine Patterns of Tension-Type Headache and Understanding Its Subgroups

**DOI:** 10.1155/2021/5544571

**Published:** 2021-09-23

**Authors:** Xinyu Hao, Fanrong Liang, Linpeng Wang, Kenneth Mark Greenwood, Charlie Changli Xue, Zhen Zheng, Ying Li

**Affiliations:** ^1^Acupuncture and Tuina School, Chengdu University of Traditional Chinese Medicine, Chengdu 610075, China; ^2^Acupuncture and Chronobiology Key Laboratory of Sichuan Province, Chengdu 610075, China; ^3^Department of Acupuncture, Beijing Traditional Chinese Medical Hospital, Capital Medical University, Beijing 100010, China; ^4^School of Social Science, University of Sunshine Coast, Queensland QLD4558, Australia; ^5^School of Health and Biomedical Sciences, RMIT University, Melbourne VIC3083, Australia; ^6^Graduate School, Chengdu University of Traditional Chinese Medicine, Chengdu 610075, China

## Abstract

Tension-type headache (TTH) is common among adults. Individualized management strategies are limited due to lack of understanding of subtypes of TTH. Chinese medicine (CM) uses the pattern differentiation approach to subtype all health conditions. There is, however, a lack of evidence-based information on CM patterns of TTH. This study aimed to identity common CM patterns of TTH. TTH sufferers were invited for a survey, consisting of a validated Chinese Medicine Headache Questionnaire (CMHQ), Migraine Disability Assessment Test, and Perceived Stress Scale. The CMHQ consisted of information about headache, aggravating and relieving factors, and accompanying symptoms. Principal component analysis was used for factor extraction and TwoStep cluster analyses for identifying clusters. ANOVA was used to compare cluster groups with disability and stress. In total, 170 eligible participants took part in the survey. The commonest headache features were continuous pain (64%); fixed location (74%); aggravated by overwork (74%), stress (74%), or mental strain (70%); and relieved by sleeping (78%). The commonest nonpain symptoms were fatigue (71%) and neck stiffness (70%). Four clusters, differing in their key signs and symptoms, could be assigned to three different CM patterns including ascendant hyperactivity of liver yang (cluster 1), dual qi and blood deficiency (cluster 2), liver depression forming fire (cluster 3), and an unlabelled group (cluster 4). Additionally, over 75% participants in clusters 1 and 2 have episodic TTH, over one-third participants in cluster 3 have chronic TTH, and a majority of participants in cluster 4 have infrequent TTH. The three patterns identified also differed in levels of disability and some elements of coping as measured with PSS. The three CM patterns identified are common clinical presentations of TTH. The new information will contribute to further understanding of the subtypes of TTH and guide the development of targeted intervention combinations for clinical practice and research.

## 1. Introduction

Tension-type headache (TTH), described as a dull, pressing, or tight quality [1], is found to be the second most prevalent chronic disorders in the world [2]. TTH is regarded as a featureless headache, as most TTH sufferers have no associated symptoms when compared with other type of headaches. Due to its nonspecific and lacking specific and distinguishing feature [3], diagnosis of TTH is, therefore, largely based on negative features [3, 4] and by excluding symptoms, syndromes, and organ diseases mimicking other primary or secondary headache [5]. However, the treatment strategies for TTH remain unspecific as its underlying mechanisms are unknown [3, 6, 7]. In addition, it is well known that people with TTH have different headache features [8–11], yet there is no standard way to subtype TTH, except for categorizing into infrequent, episodic (ETTH), and chronic TTH (CTTH) based on the frequency of headache within four weeks [1]. Such an approach does not lead to the development of effective, individualised treatment strategy [5].

Trigger factors could worsen TTH [12, 13]. Hence, identification of triggers and coping with these factors may be of values [14]. One hospital-based study confirmed that emotional stress was the major trigger factor of TTH, as outlined in other studies [8, 15]. Depression and anxiety levels in TTH sufferers have also been observed to be higher than headache-free controls [16, 17]. In addition, some triggers that are typically specific to migraine may also precipitate TTH [5], including a lack of sleep, fatigue, missing meals [18], menstruation, weather changes, relaxation after stress, exposure to bright lights, strong odors and loud noises, and ingestion of alcoholic beverages [18–20]. Presence of triggers for TTH is largely relied on patients' self-report, but has not been tested systematically [13].

Chinese medicine (CM), which has a long history of treating headaches, is viewed as “personalized medicine” as CM differential diagnostic approach guides the tailored treatment for each individual [21]. Given that TTH is not a diagnosis in CM, recognition and treatment of TTH must be based on the CM classification and common patterns of general headache. CM understands that the causes of most headaches are disorders of Qi and blood or lack of nourishment of the channels and collaterals [22]. The guiding principle of CM headache treatment is to enhance and strengthen any deficiency identified or eliminate and dispel the excessive pathogens, which vary from person to person. It is through addressing different forms of deficiency or excess that CM treatment is tailored to individuals.

Acupuncture, a key treatment modality of CM, has been reported to be effective for pain management [23, 24]. Acupuncture has been recommended as a prophylactic treatment for chronic TTH due to its effectiveness and safety profile [25, 26]. It is a valuable nonpharmacological option for patients suffering from frequent episodic or chronic TTH [27, 28]. In acupuncture treatment, each health condition is subdivided into a few common patterns based on signs and symptoms. Those patterns are important as they guide the selection of the supposed optimal individualised acupuncture protocol. Nevertheless, there is a lack of CM criteria for TTH patterns, which are important as they guide the determination of the optimal acupuncture protocol. Identifying CM patterns involves a complicated process of synthesising and analysing clinical symptoms and signs of the patient's condition to determine the location, cause, and nature of the condition [29]. Diagnosis of TTH largely relies on textbook information or expert opinion but not based on research evidence. Over the past several years, published CM studies specific to TTH in China proposed different TTH patterns based on clinical experiences and observations with some overlaps among them. Those include eleven (11) patterns of ascendant hyperactivity of liver yang, kidney deficiency, spleen deficiency, liver qi stagnation, stagnated gall bladder qi with disturbing phlegm, liver fire ascending, cold congealing in the jueyin meridian, static blood blocking collaterals, deficiency of heart and spleen, kidney yin deficiency, and deficiency of both liver yin and kidney yin [30]. Consequently, variations in the diagnosis of TTH among practitioners are common [31]. Studies have shown that it is possible to standardize and validate patterns using objective methods and evidence-based approaches [21, 32–37]. Cluster analysis has been recognized as a suitable technique to identify homogeneous subgroups for identifying CM patterns of diseases [38–40].

The aims of this study were (1) to explore CM patterns of TTH based on data collected using a validated Chinese Medicine Headache Questionnaire (CMHQ); and (2) to explore if identified CM patterns differed on information collected in modern TTH research and practice, including headache features, severity of headache-related disability assessed with Migraine Disability Assessment Test (MIDAS), and number of comorbidities, psychological profiles, such as anxiety, depression, and self-perceived level of stress. Findings of this research will lend a hand to understanding of subtypes of TTH.

## 2. Methods

### 2.1. Design

A bilingual cross-sectional survey was conducted from February 2011 to June 2012. A paper-based survey and an online survey were delivered in parallel. The online survey was performed via the SurveyMonkey® platform, whereas the paper-based survey was administrated at three sites: Melbourne, Beijing, and Chengdu. The Australian sample was from a clinical trial conducted from 2008–2012 entitled “Combined therapy of electroacupuncture and cognitive behavioural therapy for tension-type headache: a randomised controlled trial” (ANZCTR : ACTRN12608000239369) in Melbourne and from online survey. All Chinese samples were collected from two Chinese sites and through online. The two Chinese sites were of Beijing Hospital of Traditional Chinese Medicine (TCM) Affiliated to Capital Medical University and Affiliated Hospital of Chengdu University of TCM.

### 2.2. Ethics

The survey protocol was reviewed, assessed, and approved by the College Human Ethics Advisory Network of the College of Science Engineering and Health (CHEAN), RMIT University (BESHAPP10-11 HAO). The other two collaboration sites of Beijing and Chengdu were granted permission by the Department of Science Research of Beijing TCM Hospital and for the Chengdu site, by the Department of Science and Technology, Chengdu University of TCM, respectively. Those approvals were endorsed by CHEAN, RMIT.

### 2.3. Recruiting Criteria

Potential headache sufferers, aged from 18 to 65 years old, were eligible to participate if they were able to read English or Chinese; met the International Headache Society TTH diagnostic (ICHD-II) criteria of TTH or probable TTH [41]; and had one day or more of TTH attacks per month for at least one year. Exclusion criteria were TTH onset after 50 years old as those headaches are more likely to be secondary headache [42]; had more than 4 migraine attacks without aura per month, as increased attacks of migraines should be classified under migraine, rather than TTH according to ICHD-II [41]; had any migraine attack with aura per month; had been hospitalized because of the head or neck injury; or had migraine attacks which were not able to be distinguished from TTH.

### 2.4. Measurements

Demographic characteristics of the participants collected from this survey included gender, age, ethnicity, marital status, and education. Each of the listed instruments included in the survey was available in both English and Chinese versions.

Chinese Medicine Headache Questionnaire (CMHQ): the CMHQ is a symptom-based data collection tool consisting of a total 193 items which are grouped into three broad categories of pain description, aggravating and relieving factors, and accompanying symptoms. It has been used to assist CM pattern identification for headache disorders and found to be reliable and valid in capturing essential clinical indicators for making a CM diagnosis [30]. Responses to each item presented were on a 5-point Likert scale rating from 0 to 4 indicating never, seldom, sometimes, often and almost always (Appendix A).

#### 2.4.1. Migraine Disability Assessment (MIDAS) Questionnaire

The MIDAS was initially designed for the migraine population to evaluate the severity of migraine. Studies have shown it is also valid and reliable in evaluating disability associated with TTH [43–48].

#### 2.4.2. Perceived Stress Scale (PSS)

The PSS is a widely used instrument in measuring nonspecific psychological stress. Its 10-item version is among the most widely used tool to measure global perceived stress in relation to the health-related outcomes [49, 50].

#### 2.4.3. Comorbidity Checklist

A comorbidity checklist was used to assess both somatic and mental comorbidity of TTH. Development of the checklist was based on the Cumulative Illness Rating Scale (CIRS) [51] and the World Mental Health Composite International Diagnostic Interview (WMH-CIDI) [52]. The items in this checklist were reformatted in a coherent manner to detect both somatic comorbidity and the mental comorbidity.

### 2.5. Data Analysis

SPSS 18.0 was used for data analysis. A *P* value <0.05 was considered to be statistically significant. Chi-squared tests were used to examine the difference in categorical outcomes. Factor analysis and cluster analysis were conjunctively applied to obtain effective clusters and identify meaningful CM patterns for TTH. Specifically, the principal component analysis (PCA) was used for factor extraction in condensing respondents' responses to diagnostic information obtained from CMHQ items, whereas the TwoStep cluster algorithm was then used for grouping these identified factors into clusters for further evaluation [53, 54]. For PCA factor extraction, a cutoff value of 0.5 on a coefficient (“factor loading”) was adopted [55]. To determine if an identified factor was included in pattern identification based on the results from TwoStep cluster analyses, a cutoff value of 0.4 on clusters' mean scores was used. ANOVA was used to assess the cluster difference in MIDAS grades and in PSS levels. Chi-squared tests and ANOVA were employed to compare the characteristics of the resulting clusters, which enables further examination of the group differences among the CM pattern types, in MIDAS grades, and in PSS levels of the participants. Multiple comparisons were performed to compare group means via *post hoc* tests with Bonferroni correction when significant differences were observed in means across groups. For missing data handling, both case deletion and imputation methods were applied. Cases having more than 30% missing values within the total 193 items in CMHQ were deleted from the dataset, whereas cases having less than 30% missing values were remedied via the expectation-maximization algorithm [55].

**Figure 1:**
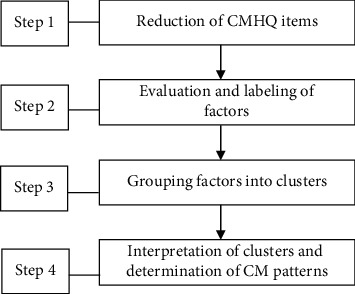
Process of CM pattern identification.

Evaluation and interpretation of data for pattern identification had four sequential steps (Figure 1). The first step was to reduce the items of CMHQ into smaller datasets using factor analysis; the second step was to assess the factors extracted and to label those factors in a clinical meaningful manner; the third step was to group (clinical meaningful) factors into clusters using cluster analysis; the final step was the identification of TTH patterns, that is, to label the clusters into clinically meaningful CM patterns. Sixteen teaching and research staff across universities and hospitals with their professional backgrounds covering CM, acupuncture, modern medicine, statistics, etc., were invited to provide their experts' opinions in the 2^nd^ and 3^rd^ steps to ensure that the labels assigned to factors and clusters were of clinical relevance and significance. Only the labels that reached 70% agreement among 16 evaluators were retained.

## 3. Results

**Figure 2:**
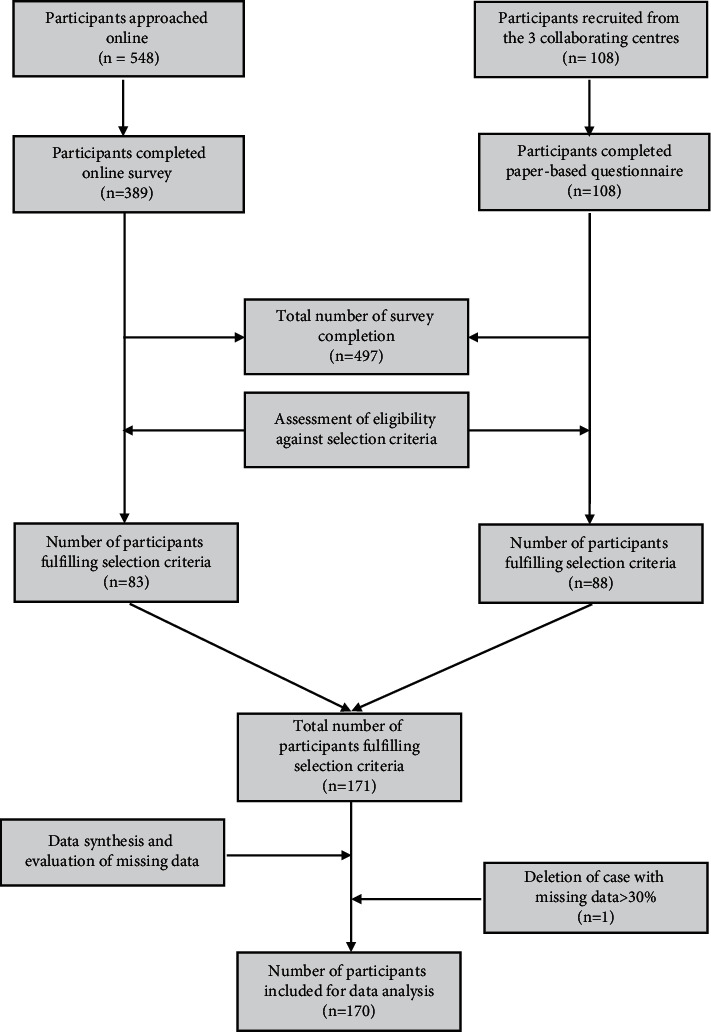
Flow chart of participant recruitment and screening process.

From February 2011 to June 2012, a total of 565 respondents took part in the survey and 497 completed it. 170 participants were eligible and included for data analysis.  Figure 2 illustrates the participant selection process. Among them, 70.6% were female and 29.4% were male (F : M = 2.1 : 1). The average age was 38 years (SD = 12). Defined by headache days per month, a majority (63%) of the included participants suffered from ETTH, whereas 23% and 14% were of CTTH and infrequent subtypes, respectively. Sociodemographic characteristics including ethnicity, marital status, and education are shown in Table 1, which indicates a majority of participants were female with a higher level of education degree in the age range of 20 to 40.

According to the CMHQ, the key features of the headaches were pain with a fixed location (74%), of continuous (66.7%) and intermittent (52.7%) nature, with tight (35.3%), heavy (34.1%), and pulsating (34.1%) sensations, and affecting the neck (61.3%) and eyes (57.2%). Overwork (74.1%), stress (73.6%), mental strain (70%), being tired (68.1%), lack of sleep (68.1%), anger or irritability (65.8%), anxiety (excessive worry) (65.5%) nervousness (56.3%), and muscular strain (muscle tightness) (53.1%) were identified as the commonest aggravating factors of headache, whereas sleeping (77.7%), medication (62.7%), lying down (62.4%), pressing on the pain area (62.1%), and massage (50.9%) were the commonest relieving factors of the headaches. Apart from headaches, neck (60%), shoulder (45.3%), and lower back (35.3%) were the most common painful areas. Of the female-related items, bright red-coloured menstrual blood (50.5%), dark-coloured menstrual blood (62.4%), headache before period (51.6%), and abdominal pain during periods (52.7%) were common referred items. Overall, the most common accompanying symptoms were fatigue (71.3%), neck stiffness (70%), and neck pain (60%).

### 3.1. TTH Pattern Identification

The exploratory analytic methods of factor analysis and cluster analysis were conjointly used given the relatively large number of CMHQ items. Firstly, PCA was used to extract factors on each part of CMHQ separately. Based on CM theory, only 41 clinical meaningful factors, including 12 factors from CMHQ part 1, 13 from part 2, and 16 from part 3, were labelled and retained for TTH pattern identification (Appendix B and C). Secondly, using the TwoStep cluster analysis, four distinct cluster groups were identified. Lastly, experts analysed the clinical characteristics of each cluster and labelled them as ascendant hyperactivity of liver yang (cluster 1), dual qi and blood deficiency (cluster 2), liver depression forming fire (cluster 3), and an unlabelled group (cluster 4) (Table 2). The first three are common patterns of headache presented in CM clinical practice.

### 3.2. Cluster Comparisons

Table 3 summarizes the characteristics of participants according to the four clusters. The four clusters differed in the aspects of demographic characteristics, stress levels, pain intensity (indicated by MIDAS item B), disability grades (indicated by MIDAS), and TTH subtypes. There were no cluster differences in gender, marital status, or education level. There was statistical age difference among the clusters (*P* < 0.001). Participants in cluster 1 were older than those in clusters 3 and 4; however, those in cluster 2 were older than those in cluster 4. Statistically significant cluster differences were also found in ethnicity distribution. Over three quarters of participants in clusters 3 and 4 were of Asian origin, but over three quarters of those in clusters 1 and 2 were of non-Asian (Oceania and European) origin.

More than half of the participants were suffering from frequency TTH in all clusters. Cluster 1 had more infrequent ETTH headache than clusters 1–3, and cluster 3 had more CTTH than the other three clusters (*P* < 0.001). ANOVA results indicated no cluster differences in the overall MIDAS scores. The level of disability, which ranged from grade I to grade IV (from low to high), was classified based on the MIDAS scores. The mean MIDAS SUM score of the current sample was 22.64 lost days, at a severe disability level (grade IV). There was a statistically significant cluster difference in the disability level (*P*=0.17). This was largely due to about 50% the participants in clusters 2 and 3 having a higher level of disability (grades III and IV), whereas 50% of cluster 4 had the lowest level of disability (grade I). There were statistically significant cluster group differences in MIDAS items 4, which indicate the reduced productivity in household because of headaches, and MIDAS B, detecting the average pain on a 0–10 scale. Post hoc *t*-tests with Bonferroni correction found clusters 2 and 4 were statistically different, with cluster 2 having more nonproductive days at home (8.2 days) due to headache and more severe headache (6.3) than cluster 4 (mean: 3 days, mean intensity: 4.7).

The average PSS score was 16.72. Compared with the normative data mean score of PSS-10 around 13 [49, 50], the existing sample had a relatively higher perceived stress than the general population. There was no cluster difference on PSS. PSS has two subscales: general distress (perceived distress; sum of items: 1, 2, 3, 6, 9, and 10) and coping ability (perceived coping; sum of items: 4, 5, 7, and 8) [56]. In this study, the average score for the “Perceived Distress” factor was 9.39, indicating a trend for a statistically significant cluster difference in this item (*P*=0.066) with cluster 4 perceiving lower level of stress. A lower score of 6.35 was observed in “Perceived Coping” factor, reflecting better coping ability. The cluster difference in this item was statistically significant (*P* < 0.001) with participants in clusters 1 and 2 coping with stress better than the other two clusters.

Comorbidities of TTH participants were calculated by counting the total number of somatic comorbidities and mental comorbidities separately. All participants had a low number of comorbidities (Table 4). There were no significant differences in somatic comorbidities among the identified four TTH clusters. Although there was no statistically significant cluster difference in mental comorbidity, cluster 4 participants reported no mental comorbidity at all (Table 3).

### 3.3. Profile of the Clusters

Table 5 illustrates the profile of the four clusters. Cluster 1 had a moderate level of pain, moderate level of disability, and moderate distribution in both physical and mental comorbidity. Participants in this cluster tended to perform the best in coping ability (PSS “Perceived Coping” factor) when compared with other three clusters. Cluster 2 had the highest pain intensity and severest disability among all four patterns. This cluster also had the largest number of participants having a physical comorbidity. Cluster 3 had a very similar pattern to cluster 2 with moderate headache intensity and severe disability. However, based on CM understanding, they differed significantly in their presentation of headache and nonpainful symptoms. In addition to the symptomatology, they were also being significantly different from their coping with stress (cluster 3 is significant among clusters, whereas cluster 2 is not). Cluster 4 was unlabelled as there were insufficient characteristics of the symptoms and signs for CM diagnosis. It had the lowest level (mild) of pain intensity and lowest disability level among the four clusters.

## 4. Discussion

### 4.1. Summary of Findings

The present study identified three distinct CM patterns of TTH through a cluster analysis of 170 TTH participants in a bilingual cross-sectional survey. The results of this study suggest that TTH can be subdivided based on symptoms and signs that are significant to the CM diagnostic process. Those clusters may or may differ in the subtypes of TTH (ETTH, frequent ETTH, and CTTH), stress level, pain intensity, and disability level. These findings expand the existing understanding of TTH symptomatology in Western medicine and TTH patterns in CM, which may help advance our understanding of the symptoms associated with TTH and subgroups of TTH as well as contribute to enhanced clinical practice in CM.

### 4.2. Pros and Cons of Explorative Analytic Methods for CM Pattern Identification

The essence of factor analysis and cluster analysis is to classify a set of observations into groups. Such an approach could be a suitable technique in supporting and verifying the CM patterns as it has been used to explore and study CM patterns in order to understand a series of diseases and conditions defined by modern medicine [57–60]. Generally, those studies identified explainable CM patterns and interpreted those modern illness/diseases in a reasonable fashion.

Although the explorative analysis could be a valuable method for the study of CM pattern identification, the results of such analysis cannot be used directly in research or clinical practice without integration with CM theory. Hence, it is necessary to incorporate experts' opinions and clinical experience in order to ensure the results being clinically meaningful. In this study, we combined the two approaches for pattern identification.

Initially, through the TwoStep cluster analysis, we identified a four-cluster solution and a five-cluster solution. Experts agreed that the patterns within the four-cluster solution tended to coincide more with the actual clinical observation and were meaningful for pattern identification. By contrast, the five-cluster solution did not lead to distinct sound/logical CM patterns. Finally, the four-cluster solution was used, resulting in three identifiable patterns and one unidentifiable cluster. However, it is likely the unidentifiable cluster including a few factors that are not powerful enough to form their own patterns. From the statistical perspective of the PCA results, we observed that the overall mean score (“power”) of the factors in cluster 4 was relatively “weak” (lower than 0.4) with most of the factor mean sores between 0.04 and 0.09. Consequently, those factors were considered having little diagnostic value for CM pattern recognition. In addition, our relatively small sample size may have restricted the number of identifiable patterns.

In PCA, the coefficient, known as the “factor loading,” refers to how strong each variable is associated with the proposed factor, is used to explain the correlation between the individual item and the overall factor [32]. As a rule of thumb, a factor loading below 0.4 indicates the loading condition is weak; 0.6, a moderate level; between 0.6 and 0.8, being large; and 0.8 or above, being very high [61]. We adopted 0.5 as the cutoff point. In contrast, in interpreting the results from TwoStep cluster analysis, the existing literature does not provide clear guiding rules for the cutoff mean score for including or excluding a factor within a cluster. In the present study, we used 0.4 of the cluster mean as the cutoff point. We then invited experts to interpret symptoms and signs and named each cluster to ensure clinical relevance. Three out of four clusters were labelled, reflecting this approach is workable (Appendix D).

### 4.3. Interpretation of Findings

The common TTH characteristics and associated symptoms identified in the present study are consistent with the findings of other studies [8–11]. The main similarities are the precipitating factors such as physical activity, stress/tension, when tired, lack of sleep, specific foods/drinks, alcohol, and skipping meals, and some accompanying symptoms such as fatigue, insomnia, and irritability. Emotion-related factors may have impacted on the presence of TTH. The present study found that stress and/or tension (73.6%) was the leading precipitating factors, and the finding is consistent with others (49.4% [8], 74.5% [62], and 63% in men and 77% [63] and 52.5% [11] in women). Only a small percentage of anxiety disorders and mood disorders were detected (7.1%, respectively). This is probably due to more than three quarters of the respondents were ETTH sufferers, as it has been shown that psychiatric comorbidities are more common in CTTH patients [64, 65], whereas those having less frequent TTH tend to have less psychiatric comorbidity [66]. Such method has been used in other exploratory research [67, 68].

Very few studies have examined the differences between ETTH and CTTH beyond headache days. In the present study, the four CM patterns differed in the TTH subtypes. The three CM patterns not only differ in headache frequency but also in headache intensity and disability. Over three quarters of participants in clusters 1 and 2 had frequent ETTH, and about one-fifth had CTTH, whereas one-third in cluster 3 had CTTH, and half had frequent ETTH. All these three clusters had very few participants with infrequent ETTH, whereas one-third of cluster 4 was having infrequent ETTH (<1 day). Those results indicate that subtypes of TTH go beyond frequency of TTH. They could differ in clinical presentation of headache as well as accompanied signs and symptoms.

We also found that, over three quarters of participants in clusters 3 and 4 were of Asian origin, but over three quarters of those in clusters 1 and 2 were of non-Asian (Oceania and European) origin. In our further analysis (Appendix E), over 40% of Asian participants were at MIDAS level 1, comparing with 20% in the non-Asian group, reflecting mild impact of TTH on function. Consistently, over one-fifth of Asian participants (22%) suffered from infrequent headache, compared with 5% of non-Asian participants did. The former also tended to have poor coping ability as assessed with PSS. Those findings are consistent with differences in four clusters identified in the current study. The ethnic difference in cluster is, therefore, likely due to the frequency and severity of headache and coping strategies, rather than differences in ethnicity. This question is, however, beyond the scope of this paper, which aims to assess if advanced statistical methods could help with TTH CM syndrome differentiation. Future studies with larger sample sizes could examine the impact of demographic features on TTH patterns.

### 4.4. Implications of the Pattern Exploration for Clinical Treatment

Currently, there is a significant gap in understanding subtypes of TTH. The IHS diagnostic criteria for TTH are designed to distinguish TTH from other types of headaches to some degree and to classify TTH into three subtypes based upon attack frequency only. Nonheadache symptoms associated with TTH are, however, not explained or accounted for. Furthermore, despite several epidemiological studies observing a series of aggravating and relieving factors and accompanying symptoms of TTH, clinical practice to date has not given adequate attention to TTH symptoms. The current study fills those gaps by using knowledge of pattern identification in CM and advanced statistical methods and identified three clinically meaningful subgroups of TTH. In addition, the identified four clusters not only differed in symptoms and signs but also in the level of disability and stress. Among them, cluster 2 had the most severe headache and highest disability level, whereas cluster 4 had mildest headache intensity, moderate disability, and was free from mental comorbidity. The presence of these subgroups of TTH indicates that there is a need to go beyond frequency of TTH, as it is possible to subcategorize TTH from a multidimensional perspective, but not just limited to the frequency of headache. Addressing headache as well as accompanying nonheadache symptoms may lead to more efficient, individualised treatment strategies. The PSS score in ascendant hyperactivity of liver yang and liver depression forming fire is high, indicating emotional stress. This needs to be acknowledged by CM practitioners. Whether CM treatment modalities are adequate for addressing those emotional difficulties is yet to be examined. In the West, psychological interventions are often used to specifically address those problems. Patients presented with either of the two patterns may require additional psychological interventions to bring out the best therapeutic effects.

### 4.5. Strengths

To our best knowledge, the present investigation is the first study using exploratory statistical method to research TTH-related symptoms as well as identifying CM patterns of TTH. Our study is the first step towards a better understanding of TTH from both CM and modern medicine aspects. This study has a few important strengths. Firstly, our method provides an alternative to current modern medicine approaches in understanding features of TTH and its subgroups, contributing essential information for future research. These results expanded the common understanding of TTH symptomatology in terms of its pain description, trigger factors, and accompanying symptoms. Secondly, our results fill the significant gap in the existing literature of CM on headaches, which there is lack of differentiation of TTH from other types of headaches, such as migraine or secondary headache. Through recruiting only TTH sufferers and using a validated questionnaire, we were able to collect comprehensive data of TTH that are of clinical significance to CM. Thirdly, the existing CM patterns of TTH in the literature relied on expert opinions alone. Our study used the evidence-based approach of combining exploratory data analysis with expert opinions to ensure the objectivity and clinical significance of our findings.

### 4.6. Limitations

There are several limitations of the current study. Firstly, the present results could be limited due to its sample size, as some other possible patterns may be observed with a larger sample size. Secondly, relying on exploratory analysis or expert opinion alone has its drawbacks. Statistically determined clusters can be affected by many factors. Expert opinions may be subjective. The present study combines both approaches to minimize this limit. Lastly, this study is a cross-sectional study, which only analyses the symptom distribution collected at a specific duration over the last 3 months. The presence and the severity of symptoms observed may change over time. Future studies may use longitudinal cohort approaches to evaluate the stability of the identified CM patterns over time and to assess the effect of interventions.

## 5. Conclusions

This study provides new and critical information for determining the symptom patterns of TTH. The finding will contribute to the subgroup or pattern classification and guide targeted intervention design, including acupuncture, for future clinical practice and research. Future studies with a large sample size will identify other patterns in addition to those reported in the current study (Tables 6–23)

## Figures and Tables

**Table 1:** Sociodemographic characteristics of the included participants.

	Frequency (*n*)	Percent
Gender (*n* = 170)		
Women	120	70.6
Men	50	29.4

Age range (*n* = 166)		
20–29	55	33.1
30–39	44	26.5
40–49	31	18.7
50–59	26	15.7
60+	10	6.0

Marital status (*n* = 165)		
Single	64	38.8
Married	77	46.7
Partnered	9	5.5
Divorced	13	7.9
Separated	2	1.2

Ethnicity distribution (*n* = 164)		
Asian	85	51.8
Oceanian	45	27.4
European	25	15.2
Arab	1	.6
Had >1 ethnicity	8	4.9

Level of education (*n* = 164)		
Postgraduate degree level	40	24.4
Graduate diploma and graduate certificate level	8	4.9
Bachelor degree level	72	43.9

Advanced diploma and diploma level	18	11.0
TAFE level	10	6.1
Secondary education	14	8.5
Primary education	2	1.2

Note: designing of the sociodemographic categories were referenced from the Australian Bureau of Statistics website (http://www.abs.gov.au).

**Table 2:** Summary of cluster characteristics according to the CMHQ data.

	Cluster 1 (*n* = 46)	Cluster 2 (*n* = 34)		Cluster 3 (*n* = 46)	Cluster 4 (*n* = 44)
Location and quality	(i) Forehead; Back of the head; Top of the head (ii) Pain quality: Throbbing; Pulsating; Pounding; Tight; A “band-like” sensation	(i) Forehead, Back of the head, Top of the head; Both side of the head (ii) Pain quality: Throbbing; Pulsating; Pounding (iii) Worse in the morning; Worse at night; All day		Whole head; No particular location	Explosive; Not dull; Sharp; Piercing

Aggravating and relieving factors	Aggravating by Dehydration; Hunger; Chocolate; Muscular strain (muscle tightness); Poor posture in sitting, standing or sleeping; Teeth grinding	Aggravating by Change of weather; Change in temperature; Hot weather; Cold weather; Dehydration; Hunger; Chocolate	Relieving by Exercise; Massage; Pressing the pain area; Warmth Coldness; Medication; Eating	Aggravating by Stress; Nervousness Irritability Excessive worry; Depression Tension or conflict-related	Aggravating by Windy days; Damp weather/Humid weather; Rainy days

Accompanying symptoms	Sensitivity to light (or to bright lights); Sensitivity to sound	“Pins and needles” or numbness in the hands and feet; Faintness; Dizziness; Watery bowel motion; Loose bowel motion		Dry mouth; Thirst; Bitter taste in the mouth	Belching; Bloating/Flatulence; Indigestion; Fear of being hot

Cluster 1: ascendant hyperactivity of liver yang. Cluster 2: dual qi and blood deficiency. Cluster 3: liver depression forming fire. Cluster 4: unlabelled group.

**Table 3:** Cluster comparisons of demographic data, TTH subtypes, MIDAS, PSS, and CIRS items.

		TTH clusters				Total (*n*)	Missing value (*n*)	*P* value^†^ Chi-square	*P* value^†^ ANOVA
		C1 (46)	C2 (34)	C3 (46)	C4 (44)	170			
Age (mean ± SD) (*n*)		45 ± 12	39 ± 11	37 ± 12	30 ± 9	143	27	N/A	≤0.001^*∗*^^05^
		44	33	37	29				

Gender (*n*)	F	35	27	30	28	120	0	.307	N/A
	M	11	7	16	16	50			

Age range (*n*)	20–29	6	8	17	24	55	4	**0.001 ** ^*∗*0125^	N/A
	30–39	10	12	10	12	44			
	40–49	10	8	6	7	31			
	50–59	14	4	8	0	26			
	60+	5	2	2	1	10			

Marriage status (*n*)	Single	13	10	16	25	64	5	0.047	N/A
	Married	22	15	25	14	77			
	Partnered	3	4	0	2	9			
	Divorced	6	4	1	2	13			
	Separated	0	1	0	1	2			

Education level (*n*)	Postgraduate	13	8	8	11	40	5	0.968	N/A
	Graduate	3	2	1	2	8			
	Bachelor	17	14	21	20	72			
	Diploma	4	5	3	6	19			
	TAFE	4	1	3	2	10			
	Secondary edu	4	4	4	2	14			
	Primary edu	0	0	1	1	2			

Ethnicity a (*n*)	Oceania	19	18	4	4	45	6	≤0.001^*∗*0125^	N/A
	European	14	7	2	2	25			
	Arab	0	0	1	0	1			
	Asian	7	5	35	38	85			
	had >1 ethnicity	5	3	0	0	8			

Ethnicity B (*n*)	Asian	7	5	35	38	85	5	≤0.001	N/A
	Non-Asian	39	28	7	6	80			

TTH subtypes (*n*)	Infrequent ETTH	1	1	7	15	24	0	≤0.001^*∗*0125^	N/A
	Frequent ETTH	36	26	23	22	107			
	CTTH	9	7	16	7	39			

MIDAS item, *n* (%)	Q1	44 (1.07)	33 (2.30)	43 (2.74)	44 (3.86)	164 (7.771)		N/A	0.408
	Q2	44 (5.34)	33 (9.67)	43 (9.81)	44 (5.50)	164 (7.43)		N/A	0.157
	Q3	44 (3.95)	33 (6.82)	43 (4.00)	44 (3.23)	164 (4.35)		N/A	0.209
	Q4	44 (6.05)	33 (8.15)	43 (4.35)	44 (2.93)	164 (5.19)		N/A	**0.038 ** ^*∗*^^**05**^
	Q5	44 (2.18)	33 (4.24))	432.65	443.86	164 (3.17)		N/A	0.606
	MIDAS a	44 (20.45)	33 (26.73)	43 (20.53)	44 (15.82)	164 (20.49)		N/A	0.259
	MIDAS B	44 (5.45)	33 (6.30)^v.4^	43 (5.42)	44 (4.68)^v.2^	164 (5.41)		N/A	**0.015 ** ^*∗*^^**05**^
	MIDAS SUM (mean score)	44 (18.59)	33 (31.18)	43 (23.56)	44 (19.39)	164 (22.64)		N/A	.310

MIDAS grade, *n* (%)	Grade I	12 (27%)	3 (1%)	14 (33%)	22 (50%)	51 (31%)		**0.017 ** ^*∗*^^**05**^	N/A
	Grade II	5 (11%)	7 (21%)	3 (7%)	4 (9%)	19 (12%)			
	Grade III	14 (32%)	8 (24%)	8 (19%)	7 (16%)	37 (23%)			
	Grade IV	13 (30%)	15 (45%)	18 (42%)	11 (25%)	57 (35%)			

PSS score (score by item)	Sum	16.68	16.19	18.79	15.11	16.72		N/A	0.092
	Perceived distress	9.85	10.06	10.22	7.52	9.39		N/A	0.066
	Perceived coping	5.04^v.3,4^	5.18^v.3,4^	7.35^*∗*^^v.1,2^	7.59^*∗*^^v.1,2^	6.35		N/A	≤**0.001**^*∗*^^**017**^

Comorbidity checklist (number of items)	Somatic comorbidity	46	34	46	44	42.9%		0.588	N/A
	Mental comorbidity	8	9	5	0	12.9%		0.060	N/A

Note 1: Australia is a county of immigration. In the section of ethnicity, the category of “had more than 1 ethnicity” indicated a group of participants in this country shares more than one ethnicity. For example, an Australian person may have his/her mother of Irish ethnicity and father of Greek. In such case, these participants may tick two options, and in data analysis, he/she was classified as participant had more than one ethnicity. Note 2: both Chi-square and ANOVA were applied to access cluster differences for comparison. Chi-square tests examine categorical outcomes, whereas ANOVA assesses the means of each cluster. *P* values correspond to comparisons between the clusters using Chi-square test or ANOVA, as appropriate. Note 3: in PSS-10, there are no cutoffs for “Perceived Distress” nor “Perceived Coping.” A lower score in “Perceived Coping” factor reflects better coping ability since the four positively stated items (4, 5, 7, and 8) in this factor are reversed scored and then summing across all items when calculating the overall score. Note 4: ^*∗*^05—the mean difference is significant at the 0.05 level; ^*∗*^0125—the mean difference is significant at the 0.0125(0.05/4) level; ^*∗*^017—the mean difference is significant at the 0.017(0.05/3) level; “*v*” denotes the clusters differed with post hoc Bonferroni correction, whereas the “*x* (figure)” after “*v*” indicates specific cluster or clusters.

**Table 4:** Summary of comorbidity checklist results.

Item		Frequency (*n*)	(%)
1	Cardiac	6	3.5
2	Vascular	11	6.5
3	Hematology	2	1.2
4	Respiratory	13	7.6
5	Ophthalmology and otorhinolaryngology	15	8.8
6	Upper gastrointestinal	16	9.4
7	Lower gastrointestinal	4	2.4
8	Hepatic and pancreatic	3	1.8
9	Renal	3	1.8
10	Genitourinary	3	1.8
11	Musculoskeletal-integumentary	13	7.6
12	Neurological	4	2.4
13	Endocrine-metabolic	6	3.5
14	Psychiatric	13	7.6
15	Female hormonal and reproductive	10	5.9
+1	Anxiety disorders	12	7.1
+2	Mood disorders	12	7.1
+3	Substance use disorders	2	1.2

**Table 5:** Relationship between patterns and other outcome measures.

Clusters	Corresponding TTH subtypes	MIDAS		Outcome measures		Comorbidity (%)	
				PSS (mean ± SD)		Physical	Mental
		Intensity (0–10)	Disability level	By PSS items (scores of item 3: the higher, the worse) (reversed scoring of item 5 and 8: the lower, the better)	By “perceived coping” factor (combined items of 4, 5, 7, and 8; the lower, the better in coping)		
Cluster 1: ascendant hyperactivity of liver yang (*n* = 46)	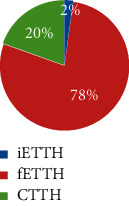	Moderate pain (5.5)	Moderate disability (grade III; 9 days)	Item 3: (2.46 ± 0.925) most often	(5.04 ± 3.025)	43.40%	17.40%
				‘*In the last month, how often have you felt nervous and “stressed”?*'	Best performance in perceived coping among clusters.		
				**Item 5: (1.39** **±** **0.755)** most often	Cluster being different from clusters 3 and 4		
				*‘In the last month, how often have you felt that things were going your way?*'			

Cluster 2: dual Qi and blood deficiency (*n* = 34)	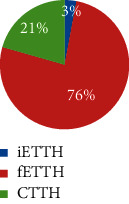	Moderate pain (6.3)	Severe disability (grade IV; 31 days)	**Item 8: (1.44** **±** **0.982)** most often	(5.18 ± 3.070)	55.90%	26.50%
				*‘In the last month, how often have you felt that you were on top of things?'*	Cluster being different from clusters 3 and 4		

Cluster 3: liver depression forming fire (*n* = 46)	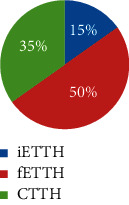	Moderate pain (5.4)	Severe disability (grade IV; 24 days)	**Item 5: (2.14** **±** **1.104)** least often	(7.35 ± 4.018)^∗^	41.30%	10.90%
				*‘In the last month, how often have you felt that things were going your way?'*	Cluster being different from clusters 1 and 2		
				**Item 8: (2.26** **±** **1.049)** LEASTLY often			
				*‘In the last month, how often have you felt that you were on top of things?'*			

Cluster 4: unlabelled group (*n* = 44)	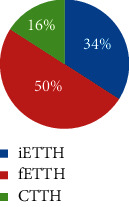	Mild pain (4.7)	Moderate disability (grade III; 19 days)	**Item 3: (1.39** **±** **0.868)** LEASTLY often	(7.59 ± 3.329)^∗^	34.10%	0.00%
				*In the last month, how often have you felt nervous and “stressed”?*	Poorest performance in perceived coping.		
					Cluster being different from clusters 1 and 2		

Note 1: in this table, “iETTH” stands for “infrequent ETTH,” whereas the “fETTH” is the abbreviation of “frequent ETTH.” Note 2: “^*∗*^”: the mean difference of PSS is significant among clusters at the 0.17 (0.05/3) level.

**Table 6:** In which areas does your headache mostly occur and how often? (Please tick (✓) one box for each item).

Location of headache	Never	Seldom	Sometimes	Often	Almost always
Forehead (front of the head)	□	□	□	□	□
Side of the head (left side)	□	□	□	□	□
Side of the head (right side)	□	□	□	□	□
Back of the head	□	□	□	□	□
Top of the head	□	□	□	□	□
Whole head	□	□	□	□	□
No particular location	□	□	□	□	□
Others (please specify)					

**Table 7:** When you have a headache, do you ever have discomfort (pain, tension, or tenderness) in the following areas? (Please tick (✓) one box for each item).

Affected area	Never	Seldom	Sometimes	Often	Almost always
Neck	□	□	□	□	□
Shoulders	□	□	□	□	□
Ears	□	□	□	□	□
Eyebrow	□	□	□	□	□
Eyes	□	□	□	□	□
Face	□	□	□	□	□
Cheeks	□	□	□	□	□
Jaw	□	□	□	□	□
Nose/bridge of nose	□	□	□	□	□
Others (please specify)					

**Table 8:** What is the sensation of your headache? (Please tick (✓) as many items as applicable).

□ Pressing	□ Explosive	□ Burning	□ Drilling	□ Cutting
□ Sharp	□ Vague	□ Heavy	□ Dull	□ Throbbing
□ Pulling	□ Empty	□ Tight	□ Distending	□ Piercing
□ Pulsating	□ Radiating	□ Pounding	□ A “band-like” sensation around the head	
Others (please specify)				

**Table 9:** When you have a headache, what is the nature of the headache? (Please tick (✓) one box for each item).

	Never	Seldom	Sometimes	Often	Almost always
Fixed (headache with fixed location)	□	□	□	□	□
Moves (headache moves around the head or shifting from side to side)	□	□	□	□	□
Continuous (constant, persistent, nonstop headache)	□	□	□	□	□
Intermittent (headache comes and goes, occasional, or periodically occurring)	□	□	□	□	□

**Table 10:** Over the last 3 months, on average, how many days per month did you have a headache? (Please tick (✓) one of the three options).

□ Less than one day	□ Between 1 and 14 days	□ 15 days or more

**Table 11:** During the course of the day, when does your headache get worse? (Please tick (✓) as many items as applicable).

□ Worse in the morning	□ Worse in the afternoon	□ Worse at the end of the day
□ Worse at night	□ All day	□ No particular time

**Table 12:** What aggravates your headache? (Please tick (✓) one box for each item).

Increased tension	Don't know	Never	Seldom	Sometimes	Often	Almost always
Overwork (e.g., prolonged working hours, long periods of studying/typing)	□	□	□	□	□	□
When tired	□	□	□	□	□	□
Mental strain (e.g., overthinking or other concentration)	□	□	□	□	□	□
Eyestrain (e.g., reading, computer, or TV)	□	□	□	□	□	□
Muscular strain (muscle tightness)	□	□	□	□	□	□
Physical labour	□	□	□	□	□	□
Lack of sleep	□	□	□	□	□	□
Poor posture in sitting, standing or sleeping	□	□	□	□	□	□

Diet						
Alcohol	□	□	□	□	□	□
Coffee	□	□	□	□	□	□
Dehydration	□	□	□	□	□	□
Hunger/being hungry	□	□	□	□	□	□
Chocolate	□	□	□	□	□	□
Cigarette smoking	□	□	□	□	□	□
Soft drink/sodas	□	□	□	□	□	□
Tea	□	□	□	□	□	□
Cheese	□	□	□	□	□	□
Dairy foods (e.g., milk, ice cream, etc.)	□	□	□	□	□	□
Monosodium glutamate (MSG)	□	□	□	□	□	□
Sugar/too much sugar	□	□	□	□	□	□
Spicy food	□	□	□	□	□	□
Overconsumption of oily food	□	□	□	□	□	□
Irregular diet (e.g., eating on the run, skip meals)	□	□	□	□	□	□

Weather						
Change of weather	□	□	□	□	□	□
Change in temperature	□	□	□	□	□	□
Exposure to bright lights or sunshine	□	□	□	□	□	□
Hot weather	□	□	□	□	□	□
Cold weather	□	□	□	□	□	□
Windy days	□	□	□	□	□	□
Damp weather/humid weather	□	□	□	□	□	□
Rainy days	□	□	□	□	□	□

Stress and emotional changes						
Stress	□	□	□	□	□	□
Nervousness	□	□	□	□	□	□
Anger or irritability	□	□	□	□	□	□
Anxiety (excessive worry)	□	□	□	□	□	□
Depression (feeling unhappy or depressed)	□	□	□	□	□	□
Tension or conflict-related (e.g., from financial constraints, family, relationship, and/or work)	□	□	□	□	□	□

Other factors						
Sneezing	□	□	□	□	□	□
Teeth grinding	□	□	□	□	□	□
Other (please specify)						

**Table 13:** What relieves your headache? (Please tick (✓) one box for each item).

	Don't know	Never	Seldom	Sometimes	Often	Almost always
Rest	□	□	□	□	□	□
Lying down	□	□	□	□	□	□
Sleeping	□	□	□	□	□	□
Medication	□	□	□	□	□	□
Exercise/light exercise	□	□	□	□	□	□
Massage	□	□	□	□	□	□
Pressing/applying pressure on the pain area	□	□	□	□	□	□
Eating	□	□	□	□	□	□
Warmth (e.g., warm environment, hot drink, hot pack, hot shower, etc.)	□	□	□	□	□	□
Coldness (e.g., cold environment, cold drink, cold pack, cold shower, etc.)	□	□	□	□	□	□
Others (please specify)						

**Table 14:** Do you have any of the following symptoms that may or may not be related to your headache? (Please tick (✓) one box for each item).

Eye-related	Never	Seldom	Sometimes	Often	Almost always

Sensitivity to light (or to bright lights)	□	□	□	□	□
Dry eyes	□	□	□	□	□
Teary eyes	□	□	□	□	□
Blurred vision	□	□	□	□	□
Sore eyes	□	□	□	□	□
Red eyes	□	□	□	□	□
Swollen eyelids	□	□	□	□	□
Eye twitching	□	□	□	□	□
Floaters in the eyes	□	□	□	□	□
Burning sensation in the eyes	□	□	□	□	□
Itchy sensation in the eyes	□	□	□	□	□

Face related (mouth, ear, and nose)					
Sensitivity to sound (or to loud noises)	□	□	□	□	□
Dry mouth	□	□	□	□	□
Thirst	□	□	□	□	□
Bitter taste in the mouth	□	□	□	□	□
Runny nose	□	□	□	□	□
Sore throat/feeling of foreign body in the throat	□	□	□	□	□
Ear discharge	□	□	□	□	□
Tinnitus (ringing in the ears)	□	□	□	□	□
Flushed face/hot red face	□	□	□	□	□

Digestion-related					
Nausea	□	□	□	□	□
Vomiting	□	□	□	□	□
Reflux	□	□	□	□	□
Belching	□	□	□	□	□
Bloating/flatulence	□	□	□	□	□
Indigestion	□	□	□	□	□
Poor appetite/loss of appetite	□	□	□	□	□

Urine and bowel related					
Yellowish urine	□	□	□	□	□
Frequent urination (especially at night)	□	□	□	□	□
Watery bowel motion	□	□	□	□	□
Loose bowel motion	□	□	□	□	□
Dry stools	□	□	□	□	□
Constipation	□	□	□	□	□

Muscle- and joint-related					
Joint stiffness	□	□	□	□	□
Neck stiffness	□	□	□	□	□
Muscle twitching	□	□	□	□	□
Weak legs and knees	□	□	□	□	□
Feeling weak in the lower back	□	□	□	□	□
Feeling cold in the lower back or lower back pain worsened by coldness	□	□	□	□	□
Cold hands and feet/cold limbs	□	□	□	□	□
Hot sensation in the palms	□	□	□	□	□
“Pins and needles” or numbness in the hands and/or feet	□	□	□	□	□

Other symptoms					
Increased forgetfulness or poor memory	□	□	□	□	□
Feeling depressed	□	□	□	□	□
Irritability/irascibility (short-tempered, easily angered)	□	□	□	□	□
Restlessness	□	□	□	□	□
Fatigue/tiredness	□	□	□	□	□
Faintness	□	□	□	□	□
Heavy sensation in the body	□	□	□	□	□
Insomnia (difficulty falling asleep or staying asleep)	□	□	□	□	□
Sighing often	□	□	□	□	□
Feverish sensation	□	□	□	□	□
Shortness of breath	□	□	□	□	□
Dizziness	□	□	□	□	□
Excessive phlegm	□	□	□	□	□
Palpitation (feeling the heart beats quickly or unusually)	□	□	□	□	□
Inability to concentrate	□	□	□	□	□
Night sweating	□	□	□	□	□
Sweating upon mild activity	□	□	□	□	□
Aversion to cold or fear of being cold	□	□	□	□	□
Aversion to hot or fear of being hot	□	□	□	□	□
Sensitive to temperature changes	□	□	□	□	□
Others (please specify)					

**Table 15:** Apart from headache, do you experience pain in any other parts of your body? (Please tick (✓) as many items as applicable).

□ Neck	□ Shoulder	□ Jaw	□ Throat
□ Ears	□ Eyes	□ Chest	□ Breasts
□ Upper back	□ Middle back	□ Lower back	□ Abdomen
□ Arms	□ Legs	□ Knees	□ Heel
□ Hips	□ Buttocks	□ Flank (side of the body)	□ Hypochondria (lower abdomen)
Others (please specify)			

**Table 16:** Information related to women's health (Only female participants need to fill in this section. This section is about women's health which may relate to your headache.).

3.3.1. Do you still have periods?	

□ No	If no, please ignore section (3.3.2) and tick the following reasons
	□ Menopause
	□ Hysterectomy
	□ Contraceptive pill
	□ Other medications
	□ Other underlying diseases
	□ Pregnancy
	□ Others (please specify)
□ Yes	If Yes, please complete the next section (3.3.2)

**Table 17:** Information related to women's health (continued) (please tick (✓) one box for each item).

	Never	Seldom	Sometimes	Often	Almost always
Irregular period cycle	□	□	□	□	□
Early periods (shortened menstrual cycle)	□	□	□	□	□
Delayed periods (prolonged menstrual cycle)	□	□	□	□	□
Light bleeding (bleeding less than normal)	□	□	□	□	□
Excessive bleeding	□	□	□	□	□
Bleeding with clots	□	□	□	□	□
Bright red-coloured menstrual blood	□	□	□	□	□
Light red-coloured menstrual blood	□	□	□	□	□
Dark-coloured menstrual blood	□	□	□	□	□
Excessive watery discharge	□	□	□	□	□
Yellow discharge	□	□	□	□	□
Headache during period	□	□	□	□	□
Headache after period	□	□	□	□	□
Abdominal pain before periods	□	□	□	□	□
Abdominal pain during periods	□	□	□	□	□
Abdominal pain after periods	□	□	□	□	□
Lower back pain before periods	□	□	□	□	□
Lower back pain during periods	□	□	□	□	□
Lower back pain after periods	□	□	□	□	□
Others (please specify)					

**Table 18:** Do you have any other diseases or health conditions diagnosed by your medical doctor? □ No. □ Yes. If yes, please tick the following listed conditions that apply to you (Please tick (✓) as many items as applicable).

□ Cardiac	Heart problem (such as cardiopathy, pericarditis, coronary heart disease, myocarditis, angina, myocardial infarction, arrhythmia, and valve problems)
□ Vascular	Circulatory problem (such as peripheral atherosclerotic disease and aneurysm of the abdominal aorta), hypertension, or cholesterol problem
□ Hematological	Blood problem (anemia, leukemia, hypercoagulability, or any other problem affecting the blood, the blood cells, the spleen, or the lymphatic system)
□ Respiratory	Respiratory problem (such as asthma, emphysema, bronchitis, pulmonary embolism, or any problems related to the lungs, bronchi, and trachea)
□ Ophthalmological and otorhinolaryngology	Problems of the eyes (such as glaucoma, cataract, and loss of vision); ears (such as important hearing impairment and otitis media); nose (such as sinusitis and rhinitis); throat (pharyngitis), and voice
□ Upper gastrointestinal(does not include diabetes)	Problems of the stomach or digestion (such as problems of the esophagus, stomach, and duodenum (such as gastritis, peptic ulcer, and duodenal ulcer)
□ Lower gastrointestinal	Intestinal problems (such as intestinal hernias, enteritis, intestinal tuberculosis, chronic diarrhea, colitis, constipation, anal problems and bowel incontinence)
□ Hepatic and pancreatic	Problems of the liver (impairment in function, liver infection, etc.), pancreas (such as pancreatitis), gallbladder (such as cholecystitis)
□ Renal	Problems of the kidneys (impairment in function, kidney infection, etc.)
□ Genitourinary	Problems of the urination system, such as ureters, bladder, urethra, prostate, and genitals (such as kidney stone, urinary incontinence, bladder infection, prostate diseases, and sexual dysfunction)
□ Musculoskeletal-integumentary	Problems of the muscles, joints, bones, connective tissue, and skin (such as fibromyalgia, rheumatoid arthritis, osteoarthritis, osteoporosis, and other forms of arthritis, carpal tunnel syndrome, Sjogren's syndrome, systemic lupus erythematosus, polymyositis etc.) and any skin (such as atopic dermatitis, eczema, and herpes) or other musculoskeletal problems
□ Neurological	Neurological (brain, spinal cord, and nerves) problem (such as cerebrovascular accident, peripheral neuropathy, facial neuritis, polyneuropathy, Gearan–Kaizer syndrome, myelitis, myasthenia gravis, and multiple sclerosis)
□ Endocrine-metabolic	Problems of the thyroid gland, obesity, diabetes, or any other hormonal problems
□ Psychiatric	Problems of depression, anxiety, alcohol, drug abuse, or other problems
□ Female hormonal and reproductive (for females only)	Problems of reproductive system (such as the uterus, ovary, and fallopian tubes) and other gynaecological problems (such as premenstrual syndrome, polycystic ovary syndrome, and pelvic inflammatory disease)
□ Others (please specify)	

**Table 19:** Have you been diagnosed by a medical doctor with any of the following mental health conditions? □ No. □ Yes. If yes, please tick the following listed conditions that apply to you.

□ Anxiety disorders	Includes anxiety, panic disorder, obsessive-compulsive disorder, posttraumatic stress disorder, agoraphobia, and social phobia
□ Mood disorders	Includes depression, mania, and bipolar (affective) disorder
□ Substance use disorders	Includes dependence or harmful use of alcohol, or drugs (opioids, sedatives, stimulants, cannabinoids, petrol, glue, etc.)
□ Others (please specify)	

**Table 20:** Rotated component matrix for CMHQ factor extraction.

CMHQ item	Part I component																
	1	2	3	4	5	6	7	8	9	10	11	12	13	14	15	16	17
1.1.1	0.755																
1.1.2			0.830														
1.1.3			0.814														
1.1.4	0.731																
1.1.5	0.673																
1.1.6		0.718															
1.1.7		0.915															
1.2.1	0.937																
1.2.2	0.928																
1.2.3																	
1.2.4		0.723															
1.2.5		0.610															
1.2.6																	
1.2.7		0.759															
1.2.8																	
1.2.9																	
1.3.1					0.555												
1.3.2		0.734															
1.3.3					0.746												
1.3.4																	
1.3.5						0.804											
1.3.6			0.577														
1.3.7																	
1.3.8							-0.570										
1.3.9		−0.584															
1.3.10						0.627											
1.3.11							0.768										
1.3.12								0.882									
1.3.13	0.789																
1.3.14			0.800														
1.3.15				0.636													
1.3.16	0.674																
1.3.17					0.640												
1.3.18	0.744																
1.3.19				0.803													
1.6.1		0.811															
1.6.2	0.774																
1.6.3	0.706																
1.6.4		0.672															
1.6.5			0.984														
1.6.6	−0.765																

CMHQ item	Part II component																
	1	2	3	4	5	6	7	8	9	10	11	12	13	14	15	16	17

2.1.1					0.816												
2.1.2					0.688												
2.1.3					0.689												
2.1.4									.								
2.1.5						0.719											
2.1.6									0.723								
2.1.7									0.607								
2.1.8						0.769											
2.1.9										0.606							
2.1.10		0.595															
2.1.11				0.771													
2.1.12				0.758													
2.1.13		0.564															
2.1.14										0.793							
2.1.15		0.585															
2.1.16		0.604															
2.1.17																	
2.1.18		0.792															
2.1.19																	
2.1.20		0.794															
2.1.21							0.698										
2.1.22							0.703										
2.1.23							0.695										
2.1.24			0.827														
2.1.25			0.838														
2.1.26																	
2.1.27			0.607														
2.1.28			0.620														
2.1.29								0.552									
2.1.30								0.592									
2.1.31								0.782									
2.1.32	0.742																
2.1.33	0.808																
2.1.34	0.828																
2.1.35	0.808																
2.1.36	0.682																
2.1.37	0.796																
2.1.38																	
2.1.39						0.507											
2.2.1	0.872																
2.2.2	0.900																
2.2.3	0.799																
2.2.4			0.772														
2.2.5		0.692															
2.2.6		0.736															
2.2.7		0.681															
2.2.8			0.655														
2.2.9		0.602															
2.2.10		0.567															

CMHQ item	Part III component																
	1	2	3	4	5	6	7	8	9	10	11	12	13	14	15	16	17

3.1.1									0.679								
3.1.2		0.573															0.528
3.1.3		0.675															
3.1.4		0.654															
3.1.5		0.636															
3.1.6		0.679															
3.1.7		0.548															
3.1.8																	
3.1.9															0.776		
3.2.10																	
3.1.11																	
3.1.12									0.718								
3.1.13					0.855												
3.1.14					0.744												
3.1.15					0.745												
3.1.16								0.605									
3.1.17								0.723									
3.1.18								0.546									
3.1.19																0.724	
3.1.20																	
3.1.21							0.704										
3.1.22							0.863										
3.1.23							0.517										
3.1.24				0.727													
3.1.55				0.748													
3.1.26				0.748													
3.1.27																	
3.1.28																	
3.1.29						0.697											
3.1.30														0.813			
3.1.31														0.743			
3.1.32											0.788						
3.1.33											0.793						
3.1.34			0.750														
3.1.35			0.694														
3.1.36			0.704														
3.1.37																	
3.1.38			0.506														
3.1.39			0.509														
3.1.40													0.724				
3.1.41																	
3.1.42												0.676					
3.1.43																	
3.1.44	0.673																
3.1.45	0.700																
3.1.46	0.798																
3.1.47	0.566																
3.1.48												0.614					
3.1.49	0.510																
3.1.50																	0.540
3.1.51	0.584																
3.1.52																	
3.1.53	0.509																
3.1.54												0.608					
3.1.55								0.537									
3.1.56																	
3.1.57	0.562																
3.1.58						0.545											
3.1.59						0.724											
3.1.60													0.731				
3.1.61										0.735							
3.1.62										0.661							
3.2.1	0.743																
3.2.2	0.792																
3.2.3			0.650														
3.2.4																	
3.2.5			0.589														
3.2.6						0.805											
3.2.7																	
3.2.8					0.766												
3.2.9																	
3.2.10																	
3.2.11	0.584																
3.2.12					0.665												
3.2.13		0.700															
3.2.14		0.778															
3.2.15																	
3.2.16			0.639														
3.2.17																	
3.2.18																	
3.2.19				0.664													
3.2.20				0.744													
3.3.2.1					0.753												
3.3.2.2				0.548													
3.3.2.3					0.810												
3.3.2.4					0.591												
3.3.2.5		0.675															
3.3.2.6	0.638																
3.3.2.7																	
3.3.2.8		0.629															
3.3.2.9	0.521																
3.3.2.10		0.805															
3.3.2.11		0.763															
3.3.2.12				0.624													
3.3.2.13				0.791													
3.3.2.14				0.699													
3.3.2.15	0.782																
3.3.2.16	0.735																
3.3.2.17			0.801														
3.3.2.18			0.660														
3.3.2.19			0.622														
3.3.2.20			0.870														

Extraction method: principal component analysis. Rotation method: varimax with Kaiser normalization.

**Table 21:** Extraction of factors.

CMHQ part 1: pain description	CMHQ part 2: aggravating and relieving factors	CMHQ part 3: accompanying symptoms
FAC1.1F1CentralHead	FAC 2.1F1Mental	FAC 3.1F1Liver-Qi&Fire
FAC 1.1F2WholeHead	FAC 2.1F2Food	FAC 3.1F2Eye
FAC 1.1F3LateralHead	FAC 2.1F3WeatherChange	FAC 3.1F3BoneJointWind
FAC 1.3F1RhythmHeadache	FAC 2.1F4NoFood&Drink	FAC 3.1F4PoorDigestion
FAC 1.3F2ExplosiveNotDull	FAC 2.1F5MentalStrain	FAC 3.1F5LiverSpleenFire
FAC 1.3F3SharpHeadache	FAC 2.1F6MuscularStrain	FAC 3.1F6YinDeficiency
FAC 1.3F4TightHeadache	FAC 2.1F7Oil&Spicy	FAC 3.1F7LiverAttackStomach
FAC 1.3F7DistendingHeadache	FAC 2.1F8WindDamp	FAC 3.1F8ENT
FAC 1.3F8EmptyHeadache	FAC 2.1F9PhysicalStrain	FAC 3.1F9LightSound
FAC 1.5F1LateOfDay	FAC 2.1F10Alcohol-DragCigar	FAC 3.1F10TemperatureSensitivity
FAC 1.5F2BothEnd	FAC 2.2F1Rest	FAC 3.1F11Constipation
FAC 1.5F3AllDay	FAC 2.2F2PhysicalStimulation	FAC 3.1F12BloodDeficiency
	FAC 2.2F3EatingRelated	FAC 3.1F13YangDeficiency
		FAC 3.1F14SpleenDeficienyOfBowel
		FAC 3.1F16Tinnitus
		FAC 3.1F17Insomnia
Included: *n* = 12	Included: *n* = 13	Included: *n* = 16

^*∗*^: In this table, “FAC” is the abbreviation of “factor,” whereas the numbers 1.X after it indicate their section number. For instance, FAC1.6F1 denotes the extracted first factor of Table 11, which summarised items of forehead, back of the head, and top of the head.

**Table 22:** Results of the cluster analysis.

Clusters	Factors (PCA mean score)		
	CMHQ part 1 factors	CMHQ part 2 factors	CMHQ part 3 factors
Cluster 1: ascendant hyperactivity of liver yang	(i) 1.3F4TightHeadache (0.68)	(i) 2.1F4NoFood&Drink (0.69)	(i) 3.1F9LightSound (0.61)
	(ii) 1.3F1RhythmHeadache (0.53)	(ii) 2.1F6MuscularStrain (0.42)	
	(iii) 1.1F1CentralHead (0.51)		

Cluster 2: dual Qi and blood deficiency	(i) 1.6F2BothEnd (0.72)	(i) 2.2F2PhysicalStimulation (0.65)	(i) 3.1F14SpleenDeficienyOfBowel (0.71)
	(ii) 1.1F3LateralHead (0.66)	(ii) 2.1F3WeatherChange (0.55)	(ii) 3.1F12BloodDeficiency (0.49)
	(iii) 1.6F3AllDay (0.65)	(iii) 2.2F3EatingRelated (0.44)	
	(iv) 1.3F1RhythmHeadache (0.56)	(iv) 2.1F4NoFood&Drink (0.42)	
	(v) 1.1F1CentralHead (0.51)		

Cluster 3: liver depression forming fire	(i) 1.1F2WholeHead (0.66)	(i) 2.1F1Mental (0.42)	(i) 3.1F5LiverStomachFire (0.51)
Cluster 4: nonspecific cluster	(ii) 1.3F2ExplosiveNotDull (0.29)	(ii) 2.1F8WindDamp (0.08)	(ii) 3.1F4PoorDigestion (0.07)
	(iii) 1.3F3SharpHeadache (0.04)		(iii) 3.1F10TemperatureSensitivity (0.09)
			(iv) 3.1F11Constipation (0.04)

^*∗*^: this table reports the results of the TwoStep cluster analyses. Computed from the TwoStep cluster algorithm, mean scores of extracted factors that exceeded value 0.4 are listed for clusters 1, 2, and 3. The cutoff of 0.4 was determined as those clusters being considered to be clinically relevant.

**Table 23:** Group comparisons on MIDAS, PSS, and TTH subtypes between Asian and Non-Asian participants.

MIDAS	Asian vs. Non-Asian			Chi	*t*-test
	MIDAS LEVEL			0.007^*∗*^	N/A
	MIDAS SUM			N/A	0.122
	MIDAS a (item)			N/A	0.073
	MIDAS B (item)			N/A	0.000^*∗*^

**PSS**	**Asian vs. Non-Asian**			**Chi**	***t*-test**
	PSS SUM			N/A	0.615
	PSS perceived distress			N/A	0.445
	PSS perceived coping ability			N/A	0.000^*∗*^

**TTH subtypes**	**Asian**	**Non-Asian**	Total	**Chi**	***t*-test**
(1) Infrequent ETTH	19	4	23	0.005^*∗*^	N/A
(2) Frequent ETTH	47	56	103		
(3) Chronic TTH	18	20	38		
Total	84	80	164		

^*∗*^: the mean difference is significant at the 0.05 level.

## Data Availability

The datasets used and/or analysed during the current study are available from the corresponding author on reasonable request.

## Abbreviations

ANOVA: One way analysis of variance
CIRS: Cumulative Illness Rating Scale
CM: Chinese medicine
CMHQ: Chinese Medicine Headache Questionnaire
CTTH: Chronic tension-type headache
ETTH: Episodic tension-type headache
ICHD-II: Headache Society TTH Diagnostic Criteria (2nd edition)
IHS: International Headache Society
MIDAS: Migraine disability assessment test
PCA: Principal component analysis
PSS: Perceived Stress Scale
SD: Standard deviation
TCM: Traditional Chinese medicine
TTH: Tension-type headache
WMH-CIDI: World Mental Health Composite International Diagnostic Interview.

## Appendix

### A. Chinese Medicine Headache Questionnaire (CMHQ)

Please tick the listed items that best describe your headache and other symptoms you experienced over the last 3 months.
